# Draft Genome Sequence of “Candidatus Methylacidiphilum kamchatkense” Strain Kam1, a Thermoacidophilic Methanotrophic Verrucomicrobium

**DOI:** 10.1128/genomeA.00065-15

**Published:** 2015-03-05

**Authors:** Helge-André Erikstad, Nils-Kåre Birkeland

**Affiliations:** a Department of Biology, University of Bergen, Bergen, Norway; b Centre for Geobiology, University of Bergen, Bergen, Norway

## Abstract

“Candidatus Methylacidiphilum kamchatkense” strain Kam1 is an aerobic methane-oxidizing thermoacidophilic bacterium belonging to the *Verrucomicrobia* phylum. It was recovered from an acidic geothermal site in Uzon Caldera, Kamchatka, Russian Federation. Its genome possesses three complete *pmoCAB* gene clusters encoding particulate methane monooxygenase enzymes and a complete Calvin-Benson-Bassham cycle for carbon assimilation.

## GENOME ANNOUNCEMENT

Methane-oxidizing bacteria are capable of using methane as the sole carbon and energy source and are important sinks for limiting the release of methane to the atmosphere. The use of methanotrophs as biocatalysts for the conversion of natural gas to biofuels, biopolymers, single-cell proteins, and high-value products is a promising approach for biotechnological use of natural gas (1). Large-scale methane fermentation at a commercial scale has already been proven as a technically viable process using a loop reactor system (http://calysta.com/company).

“Candidatus Methylacidiphilum kamchatkense” strain Kam1, is a rod-shaped and Gram-negative aerobic methane-oxidizing bacterium belonging to the *Verrucomicrobia* phylum, isolated from an acidic hot spring in the Uzon Caldera, in Kamchatka, Russian Federation (2), and was one on the first nonproteobacterial methanotrophs to be described. Two related strains, “*Ca.* Methylacidiphilum infernorum” (strain V4) and “*Ca*. Methylacidiphilum fumariolicum” (strain SolV), have also been recovered from similar geothermal environments (3, 4), and together they form a novel verrucomicrobial subdivision (5). All three isolates are thermoacidophilic, with optimal growth at pH 2 to 3.5 and 55 to 60°C. The draft genome of strain Kam1 was sequenced with 454 technology using a GS-FLX pyrosequencer at GATC Biotech, Germany (http://www.gatc-biotech.com). A total of 300,132 reads accounting for 72,788,864 bases were obtained. Assembly into 41 contigs was done with Newbler version 2.9 accessed through the Lifeportal, University of Oslo (http://www.uio.no/english/services/it/research/hpc/lifeportal).

The draft genome sequence of “*Ca*. Methylacidiphilum kamchatkense” Kam1 comprises 2,210,643 bp in size, with a GC content of 40.8% and 1,805 protein-coding sequences. A single rRNA operon is present, together with 46 tRNA genes. This is comparable with the genome of the other strains, V4 and SolV, whose complete genomes constitute ~2.3 and 2.5 Mbp, respectively (6, 7). An average nucleotide identity (ANI) analysis using the online ANI calculator (http://enve-omics.ce.gatech.edu/ani/index) revealed ANI values of 78 and 93%, respectively, when the Kam1 draft sequence was compared with the complete sequences of strains V4 and SolV. This indicates that Kam1 represents a separate species, as this value is lower than the threshold value of 95%, which corresponds to a genomic DNA:DNA hybridization value of 70% and is a common threshold value for distinction between species (8).

Three operons, each encoding one set of the three subunits of the key enzyme, particulate methane monooxygenase, *pmoCAB1-3*, as well as one truncated *pmoCA* cluster and a solitary *pmoC* gene, have been described before (9). The ribulose monophosphate pathway and the serine pathway were until recently believed to be the main carbon assimilatory routes in aerobic methanotrophs. Like strains V4 and SolV, strain Kam1 lacks complete serine and ribulose monophosphate pathways for assimilation of carbon from formaldehyde, while possessing a complete Calvin-Benson-Bassham cycle, confirming an autotrophic type of carbon assimilation also in this species (10). Genes for the Embden-Meyerhof-Parnas glycolytic pathway, the pentose phosphate pathway, and the tricarboxylic acid cycle are present.

### Nucleotide sequence accession numbers.

This whole-genome shotgun project has been deposited at DDBJ/EMBL/GenBank under the accession number JQNX00000000. The version described in this paper is version JQNX01000000.

## References

[1] Fei Q, Guarnieri MT, Tao L, Laurens LM, Dowe N, Pienkos PT 2014 Bioconversion of natural gas to liquid fuel: opportunities and challenges. Biotechnol Adv 32:596–614. doi:10.1016/j.biotechadv.2014.03.011. 24726715

[2] Islam T, Jensen S, Reigstad LJ, Larsen O, Birkeland NK 2008 Methane oxidation at 55°C and pH 2 by a thermoacidophilic bacterium belonging to the *Verrucomicrobia* phylum. Proc Natl Acad Sci USA 105:300–304. doi:10.1073/pnas.0704162105. 18172218 PMC2224206

[3] Dunfield PF, Yuryev A, Senin P, Smirnova AV, Stott MB, Hou S, Ly B, Saw JH, Zhou Z, Ren Y, Wang J, Mountain BW, Crowe MA, Weatherby TM, Bodelier PL, Liesack W, Feng L, Wang L, Alam M 2007 Methane oxidation by an extremely acidophilic bacterium of the phylum *Verrucomicrobia*. Nature 450:879–882. doi:10.1038/nature06411. 18004300

[4] Pol A, Heijmans K, Harhangi HR, Tedesco D, Jetten MS, Op den Camp HJ 2007 Methanotrophy below pH 1 by a new *Verrucomicrobia* species. Nature 450:874–878. doi:10.1038/nature06222. 18004305

[5] Op den Camp HJ, Islam T, Stott MB, Harhangi HR, Hynes A, Schouten S, Jetten MS, Birkeland NK, Pol A, Dunfield PF 2009 Environmental, genomic and taxonomic perspectives on methanotrophic *Verrucomicrobia*. Environ Microbiol Rep 1:293–306. doi:10.1111/j.1758-2229.2009.00022.x. 23765882

[6] Anvar SY, Frank J, Pol A, Schmitz A, Kraaijeveld K, den Dunnen JT, Op den Camp HJ 2014 The genomic landscape of the verrucomicrobial methanotroph *Methylacidiphilum fumariolicum* SolV. BMC Genomics 15:914. doi:10.1186/1471-2164-15-914. 25331649 PMC4210602

[7] Hou S, Makarova KS, Saw JH, Senin P, Ly BV, Zhou Z, Ren Y, Wang J, Galperin MY, Omelchenko MV, Wolf YI, Yutin N, Koonin EV, Stott MB, Mountain BW, Crowe MA, Smirnova AV, Dunfield PF, Feng L, Wang L, Alam M 2008 Complete genome sequence of the extremely acidophilic methanotroph isolate V4, *Methylacidiphilum infernorum*, a representative of the bacterial phylum *Verrucomicrobia*. Biol Direct 3:26. doi:10.1186/1745-6150-3-26. 18593465 PMC2474590

[8] Goris J, Konstantinidis KT, Klappenbach JA, Coenye T, Vandamme P, Tiedje JM 2007 DNA-DNA hybridization values and their relationship to whole-genome sequence similarities. Int J Syst Evol Microbiol 57:81–91. doi:10.1099/ijs.0.64483-0. 17220447

[9] Erikstad HA, Jensen S, Keen TJ, Birkeland NK 2012 Differential expression of particulate methane monooxygenase genes in the verrucomicrobial methanotroph “Methylacidiphilum kamchatkense” Kam1. Extremophiles 16:405–409. doi:10.1007/s00792-012-0439-y. 22488571

[10] Khadem AF, Pol A, Wieczorek A, Mohammadi SS, Francoijs KJ, Stunnenberg HG, Jetten MS, Op den Camp HJ 2011 Autotrophic methanotrophy in Verrucomicrobia: *Methylacidiphilum fumariolicum* SolV uses the Calvin-Benson-Bassham cycle for carbon dioxide fixation. J Bacteriol 193:4438–4446. doi:10.1128/JB.00407-11. 21725016 PMC3165502

